# Setting up a temporary isolation tent site for asymptomatic COVID-19 positive male migrant workers in Qatar

**DOI:** 10.5339/qmj.2022.55

**Published:** 2022-11-15

**Authors:** Guillaume Alinier, Anan K A Al Badawi, Ramy Z E M Gharib, Sunil Ramsumar, Brendon D Morris

**Affiliations:** ^1^Hamad Medical Corporation Ambulance Service, Doha, Qatar Email & ORCID ID: galinier@hamad.qa & https://orcid.org/0000-0003-4255-4450; ^2^Weill Cornell Medicine-Qatar, Doha, Qatar; ^3^School of Health and Social Work, University of Hertfordshire, Hatfield, UK; ^4^Faculty of Health and Life Sciences, Northumbria University, Newcastle upon Tyne, UK

**Keywords:** COVID-19, Isolation, Facilities, Infection control, Temporary, Tent site

## Abstract

Background: Qatar has a unique demographic composition, involving hundreds of thousands of male blue-collar workers living in places where physical distancing measures are difficult to implement. This study aimed to describe the rapid development and operations of a temporary isolation facility, which was composed of tents, for asymptomatic COVID-19 positive migrant workers.

Design: The government established several temporary isolation facilities to house this important group of the community. This was achieved through daily meetings over a short period, thanks to the collaboration of government and private partners, in parallel to the facility being built and required resources procured.

Results: A 3,726-patient capacity isolation facility composed of large tents was constructed in 1 month and was kept operational from April 16 to June 20, 2020. Over that period, it received a total of 18,900 patients. It took 10 days from the decision to set up the first part of the isolation facility to admitting its first occupants.

Conclusions: The COVID-19 pandemic necessitated the implementation of unprecedented global public health and physical distancing measures to contain the spread of the virus among the population. Rapidly opening a temporary COVID-19 isolation facility bought the healthcare sector time to set up more permanent solutions to contain the spread of the virus.

## Introduction

In December 2019, China declared an outbreak of pneumonia of unknown origin in Wuhan City. This new disease, which mainly affects the respiratory system like the severe acute respiratory syndrome (SARS) and the Middle East respiratory syndrome (MERS),^1^ was determined to be caused by severe acute respiratory syndrome-coronavirus 2 (SARS-CoV-2). It was initially named 2019-nCov, i.e., novel coronavirus disease, and was then officially called coronavirus disease 2019 (COVID-19). The World Health Organization (WHO) declared the COVID-19 pandemic on March 11, 2020.^2^ Infected patients might exhibit mild to severe symptoms. However, it can be fatal, primarily in geriatric patients, including those with comorbidities such as cardiovascular diseases and diabetes mellitus.^3^ COVID-19 is also increasingly described as a vascular disease in the longer term, often post-recovery.^4^

The global response to this pandemic was to establish facilities to isolate patients with COVID-19 from the community, recommend physical distancing, implement the use of a contact tracing mobile application, close mass-gathering places, replace face-to-face by distance education, and direct people to work from home when possible. These measures were recommended to flatten the curve of the spread of the virus among the population and were also implemented in Qatar.^5-7^

Being the first country affected by COVID-19, China built 16 temporary hospitals in Wuhan City in 3 weeks to accommodate 13,000 mild-to-moderate cases in response to the bed crisis in COVID-19 hospitals. They were mostly “Fangcang shelter hospitals,” which were public venues such as stadiums and exhibition centers converted into healthcare facilities. These new hospitals were essential to isolate confirmed COVID-19 cases from the community, as they were cost effective, rapidly built, and could have a large bed capacity. These facilities provided necessary medical care, food, and social activities to all admitted patients.^8^

The State of Qatar recorded the first confirmed case of COVID-19 on February 27, 2020,^9^ and imposed various restrictions recommended by the WHO.^10-12^ To limit virus spread, the government implemented several measures, restricting mass gatherings including closing places such as cinemas and banquet halls.^7^ They also implemented distance learning, and many employees were advised to work from home.^11^ The mandatory use of a contact tracing mobile application (Ehteraz, from the Arabic word meaning “precaution” or “be careful”) was implemented for everyone to use when they stepped out of their house. Many testing facilities opened, and care was freely provided to patients with COVID-19.^12-15^

With nearly 2.8 million inhabitants, Qatar has quite a unique demographic mix. It comprises a predominantly young male expatriate workforce employed in infrastructure, service, and hospitality industries and hence play a significant role in the development of the country.^16^ Most migrant workers live in shared accommodations where physical distancing is not possible; thus, COVID-19 has significantly affected this important population group, especially those vulnerable because of pre-existing comorbidities.^17^

However, asymptomatic individuals do not warrant hospitalization, remain undetected if not tested, and infect others. Other solutions needed to be rapidly developed to prevent disease spread among the workers’ population while not depleting hospital beds.^18^ Thus, the rapid development of temporary isolation facilities in North Doha came into play starting in March 2020 alongside the urgent need to assign or build facilities to isolate confirmed cases in hospitals, hotels, compounds, or camp-like settings under direct observation of a medical team. The Hamad Medical Corporation Ambulance Service (HMCAS) played a key role in leading the operations of most of these facilities, in addition to other isolation facilities designated for citizens, professionals, and families, where they could be accommodated free of charge.

This study aimed to report the development and operations of one of the largest temporary isolation facilities for COVID-19-positive male blue-collar workers in the State of Qatar.

### A national project for COVID-19 isolation facilities

Under the leadership of the National Health Strategic Command Group on COVID-19, key stakeholders representing various government and private entities were asked to rapidly establish isolation facilities for male and female workers with confirmed COVID-19 because they developed symptoms and self-referred themselves for testing, had recently traveled, or were invited for testing because of contact tracing. This important project demonstrated how crisis resource management (CRM) principles could be implemented to address a critical issue, as teamwork and communication were key ingredients.^19,20^ Various stakeholders constituted a working group chaired by a senior HMCAS senior leader (BM), and available resources were assessed and mobilized. To demonstrate corporate social responsibility, the private sector was invited to make donations (beds, linen, and other items) some of which could be individually issued to patients. There was an ongoing process of re-evaluating the situation and objectives and anticipating how the situation could evolve.

Recently built and unoccupied worker accommodations were immediately reconfigured as isolation camps with a total capacity of just over 4,000 beds (for male and female patients). In anticipation of a significant surge in the number of COVID-19 cases, the overall capacity had to be increased by setting up an additional temporary isolation facility. This was in parallel to other projects also setting up isolation and quarantine facilities in apartment blocks, compounds, and hotels, with an additional total capacity exceeding 29,000 beds to accommodate citizens, professionals, and families. By mid-May 2020, the overall capacity reached over 37,000 beds when there were approximately 35,000 active COVID-19 cases in Qatar.^21^ Adding to that capacity, and if they had adequate facilities at home (bedroom with en suite bathroom), some patients who were asymptomatic were allowed to self-isolate at home and were remotely monitored daily by a medical team through telephone consultations. Additionally, HMC seconded and deployed many healthcare staff from the private sector and assigned them to isolation facilities.

### Setting up a large capacity isolation facility for blue-collar workers

A government plot of land approximately 25 km north of Doha was provided to the Ministry of Public Health to build a temporary isolation camp constituted of large air-conditioned tents for asymptomatic or mildly symptomatic COVID-19 male migrant blue-collar workers. Daily meetings were organized with all stakeholders at the same time the site was being prepared with proper water and electrical services, and concrete foundations and tents were being erected. Various experts were involved to determine the exact services required, security and safety measures to implement, and how the facility would be operated and managed.

**Figure 1.**
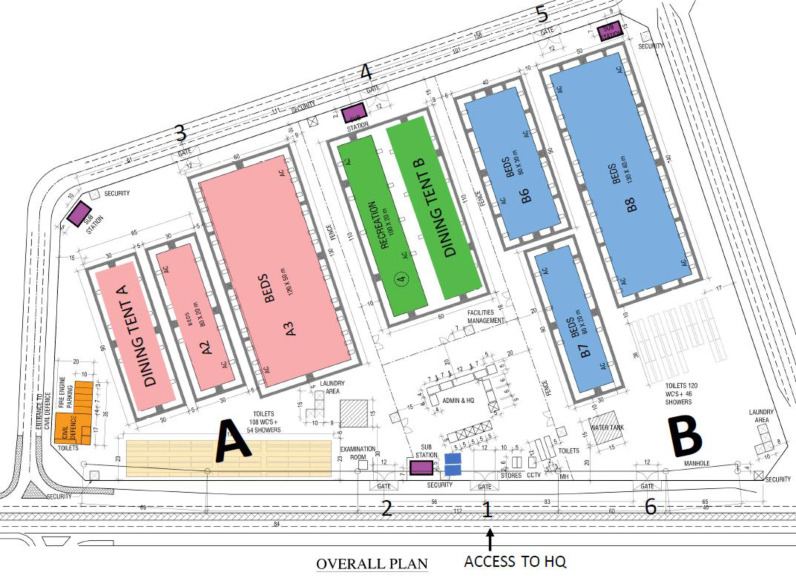
Layout of the temporary isolation tent camp.

Within 10 days, the first part was handed over and ready to receive patients (Figure 1, zone A). It included dining and recreational areas, sleeping quarters, laundry, toilet, and shower facilities. The second part opened 1 month later, which increased the capacity from 1,420 beds to 3,726 beds. Facility operations were fully funded by the government. The overall temporary isolation tent camp diagram is shown in Figure 1. 

### Zones and facilities

Owing to the facility’s size and to facilitate access to medical and public services, it was divided into zones A and B. Zone A contained two large tents used as sleeping quarters, with a combined capacity of 1,420 patients. Zone B comprised three large sleeping tents and could accommodate a total of 2,306 patients. Both zones had an adjacent area, with a combined total of 228 toilets and 100 showers, and laundry facilities so patients could do their washing. Each was assigned a bed with a number to assist in identifying their location and facilitating admission and discharge from the facility. Each patient had a locker and complimentary Wi-Fi. Owing to the high temperature and humidity at most times of the year, all facilities were air-conditioned. Each zone contained a dining tent where three daily meals were served; however, patients shared a common recreational area on a rotational basis. The patients were briefed about the restaurant and recreational area access timing during their admission based on their bed number. Since patients were from different nationalities, various food types were available to suit their preference. As an infection control measure, all facilities were regularly disinfected.

### Security and safety

Since this facility was designated to house thousands of patients, ensuring their safety, welfare, and protection and that of the staff was critical. A fire station, facility management, maintenance office, clinic, and security posts with a closed-circuit television office were set up within the camp.

Numerous police units outside and inside the facility played a critical role in policing patients’ behavior, preventing potential riots, assisting medical staff in doing their work smoothly, and providing invaluable assistance during the patients’ discharge process. The police presence around the facility also dissuaded any unauthorized person from entering and prevented patients from leaving the facility before they were properly discharged.

As shown in Figure 1, the facility had six gates, each with a specific purpose, i.e., patient admission, medical staff entrance, and deliveries, and they were manned by at least two security personnel. Additional security staff were stationed inside the facility to manage any potential situation and provide assistance to patients. All police officers, security, and housekeeping personnel received training regarding infection control and first aid. This training was conducted regularly by HMCAS staff.

### Entertainment, well-being, and mental health

A recreational area of 2,000 square meters provided patients with multiple activities to improve their well-being and keep them entertained. It included table tennis, basketball, volleyball, drawing, and card playing. Drawing was run as an art therapy workshop and was shown to improve participants’ well-being, as it reduced patients’ feeling of isolation, loneliness, and depression.^22^ Tablets with Internet access enabled patients to communicate with their families and reassure them about their health and well-being. Praying and movie theater areas were also accessible.

The effect of isolating patients with COVID-19 from the community has been documented in the literature. This social isolation can result in chronic loneliness and boredom, which, if prolonged, may be detrimental to one’s physical and mental health. Crisis often significantly affects the human psyche, heightening danger alert, and exacerbating anxiety.^23^ Physical inactivity and sedentary behavior while in isolation can increase the risk of cardiovascular diseases. A study conducted in Qatar in another isolation facility with symptomatic COVID-19 male workers and fewer entertainment options identified a high prevalence of depressive and anxiety symptoms among its occupants.^24^

### Call center service

Owing to the size of the isolation facility, communication could be difficult, so a call center was established to handle patients’ requests such as laboratory or swab results, expected discharge date, and delivery of personal items and to report any emergency. This service was available 24/7 for all patients.

### Clinical staff

Since the project was designed to accommodate a large number of patients, recruitment of a sufficient number of clinical staff was needed to meet patients’ needs. The facility management employed 12 nurses responsible for patient admission and bed allocation, providing medication in the clinics and responding to those requiring any medical assistance if they requested support or were referred to the facility’s clinic. Four physicians were responsible for patients’ physical examination during the admission phase and for ensuring their eligibility to be admitted to this facility, which was mainly for patients who were mainly asymptomatic and did not require special assistance. Additionally, physicians ensured the adequacy of patients’ maintenance medications of their potentially pre-existing chronic illnesses, addressing any new health issues, providing health education, including COVID-19 symptoms, modes of transmission, and safety precautions, and requesting the transfer of those in need of hospital care. Finally, they were involved in patients’ physical examination before discharge and prepared the daily discharge lists. In addition, several other staff and volunteers served in administrative and other supportive roles.

### Rapid response team

Each shift had a team consisting of a doctor, nurse, and paramedic responsible for responding to any medical emergency within the facility. Generally, nurses were not permitted to perform any intervention without a physician’s order, so they were trained by the Ambulance Service supervisor on how to approach and manage sick or injured patients using HMCAS equipment. This team was activated if a call was placed to the call center or patients contacted security personnel for a medical emergency.

### Patient flow from admission through to discharge

#### Triage

All patients sent to the facility were triaged at a nearby facility based on several factors including age, past medical history, present medical condition, and multiple tests including physical examination, vital signs, chest X-ray findings, blood investigations, and electrocardiogram. After the triage process, their final disposition was decided. Those who were symptomatic but with body weakness or were >65 years of age were transferred to hospital by ambulance for additional medical attention; those who were >55 years old, asymptomatic, with or without comorbidities, were admitted to another isolation facility. Those < 55 years old were transported to the tent isolation facility. The triage facility managed on average 600 patients per day in the period from March to October 2020.

#### Admission

After triage, the charge nurse made a list of patients to be admitted to the tent isolation facility. In collaboration with a HMCAS representative, the nurse loaded the patients onto buses and verified their identity before their transfer to the tent site. The HMCAS representative was responsible for coordinating with the HMCAS supervisor at the other site regarding the bus arrival time.

On bus arrival, security personnel separated patients into lines and inspected their bags for prohibited items such as tobacco, lighters, and sharp objects. Then, with the assistance of a HMCAS representative, one nurse verified patients’ identity and oriented them to the facility, its services, and its rules and regulations. Finally, nursing staff assisted patients in finding their assigned bed. All patients were given a personal item bag containing slippers, pyjamas, phone charger, and other items. The maximum number of patients being admitted to the tent isolation facility in a single day reached 900 on May 15, 2020.

#### Discharge

The administrative team prepared the discharge list each morning and sent it to the doctors’ team. Doctors examined patients’ records to ensure they were eligible to leave the facility. Thereafter, doctors conducted a clinical examination of these patients to ensure they were symptom-free. Doctors had the right to keep some patients in the facility for additional days if their health condition was unsatisfactory.

The doctors communicated the final list to the nursing staff and on-duty HMCAS supervisor who oversaw the discharge process in collaboration with their assistants. The HMCAS supervisor was in charge of the buses that transported patients to their home and the security staff that organized patients’ movement. After discharge, a list of patients who had physically exited the facility was produced to keep the system updated. The maximum number of patients discharged in a single day reached 200 on May 27, 2020.

### Duration of stay

The primary goal of isolation is to slow disease spread among the population. Since the isolation period that ensured patients were not at risk of transmitting the disease to the community was initially undetermined, several protocols pertaining to the minimum required patients’ length of stay in the facility were sequentially implemented as knowledge was gained around the virus. These protocols were developed in accordance with WHO recommendations and the best available evidence published at the time.

The protocol enforced in other isolation facilities at the time was implemented in the temporary tent isolation facility as it opened on April 16, 2020, and required the patient to remain in the facility for 21 days following the initial positive reverse transcription-polymerase chain reaction (RT-PCR) test and be tested again.^25^ If the swab sample was negative, another swab was taken the following day. If the result was negative again, the patient could be considered for discharge. If that second swab result was positive, the patient stayed for another 7 days, and the cycle was repeated until two consecutive negative swabs were obtained.

The second protocol was implemented at the end of April 2020, which required patients to stay in the facility for 21 days following the initial positive RT-PCR test, and no re-testing was performed. It was implemented based on recent research suggesting that 89% of viral ribonucleic acid was eliminated within 21 days of the disease manifestation.^25^ Another study conducted in China found that isolating patients with COVID-19 for 17–21 days effectively reduced the transmission risks.^26^

The third protocol was established early June in response to a WHO recommendation regarding the discharge of patients with COVID-19 from isolation facilities. Patients were discharged only if they were found to be symptom-free for a 3-day period following 10 days after a negative swab sample. Evidence shows that patients were then unlikely to be able to transmit the virus to others.^27^ However, to ensure community safety, all discharged patients were requested to sign an agreement to continue self-isolation at home for another 7 days.

## Discussion

### Ems And Hmcas Role

Globally, emergency medical services (EMS) are responsible for the prehospital assessment, treatment, and transportation of patients irrespective of whether they might have COVID-19 or not, thereby complementing the work of the hospital system.^28-31^ These functions institute further benefits by facilitating improved pandemic planning, management, and transportation of the sick and injured from the community to an appropriate healthcare or other designated facility. During an epidemic or pandemic outbreak, EMS providers should continue these tasks, in addition to identifying people who are at risk for potential pandemic-associated disease or health complications.^28,32,33^ The COVID-19 outbreak provided a rare opportunity to expand on this area given its global occurrence, highly infectious nature of the virus, and extreme societal interventions implemented to reduce its spread while taking care of patients with more or less severe symptoms.

### Process progress

Consequently, understanding the role and utilization of these services during a pandemic is important. However, research regarding this is lacking, or it is poorly described in the literature. It is limited to descriptions of small samples, narrow in scope, or focused on localized outbreaks. In contrast to our experience as EMS providers in Qatar, which is quite unique, other than reports containing recommendations,^17^ there is limited information to learn how to set up and operate a large temporary isolation facility. HMCAS demonstrated great adaptability and resilience and played a prominent role by treating and transporting confirmed and suspected COVID-19 cases to places designated for them and even responding to an increasing number of emergency calls that were not directly related to COVID-19. Aside from its active role in conducting swab tests and contribution to COVID-19 vaccination campaigns,^14^ HMCAS has constantly been planning to face such difficult situations; thus, they were well prepared to respond whenever the need arose. To prevent further disease transmission in the community, there was an urgent need to assign or build facilities to isolate confirmed cases either in hospitals or in camp-like settings under direct medical observation. HMCAS played a key role in managing these isolation facilities. The temporary tent isolation facility described above remained operational from April 16 to June 20, 2020, and received a total of 18,900 patients. No major concerns or incidents were reported, but weather conditions (rising temperature and wind), gradual deterioration of the facilities (damages caused by patients and wind, overworked air-conditioners causing occasional failures, etc.), and opening of more appropriate buildings to be used as isolation facilities while maintaining the required patient capacity, lead to the closure of the tent site on June 20, 2020. If a similar setup needs to be rapidly implemented, we recommend that each tent should be built with a smaller holding capacity to make patient management easier and cooling more effective. Some medical challenges faced were related to many patients not having pre-existing electronic medical records because they had never been treated at one of the government’s healthcare facilities. Generally, there was a lack of manpower, as all healthcare facilities were stretched well over their normal operational capacity, and many staff took sick leave because of COVID-19 in most cases.^13^ It was a fast-moving environment, and being a temporary facility, the resources were relatively limited.

## Conclusion

Collaboration among various key health, governmental, and industry stakeholders was critical to the successful and expedited implementation of this temporary isolation facility in the State of Qatar for patients with COVID-19. Numerous lessons were learned, that is, to respond effectively to any future pandemic. A key aspect, related to the CRM principles, was to remain flexible and adapt to a constantly evolving situation, maintain situational awareness, working according to the WHO recommendations while exercising critical thinking to consider the local context and type of patients cared for. The temporary tent site was a relatively effective solution to rapidly set up a large capacity isolation facility for mostly asymptomatic COVID-19 male migrant workers while other more permanent facilities could be prepared.

### Disclaimer

The views presented in this manuscript are those of the authors and do not necessarily represent the views or official policy of Hamad Medical Corporation.

### Author contributions

Study design: GA, AKAAB, and BDM

Data acquisition: RZEMG and SR

Manuscript writing: GA and AKAAB

Critical review and major scientific input: GA, AKAAB, RZEMG, SR, and BDM

### Conflict of interest

The authors have no conflict of interest to declare.

### Funding

There was no funding for this study.

### Ethical approval

Not applicable.
